# Long‐term outcomes of proton therapy for prostate cancer in Japan: a multi‐institutional survey of the Japanese Radiation Oncology Study Group

**DOI:** 10.1002/cam4.1350

**Published:** 2018-02-14

**Authors:** Hiromitsu Iwata, Hitoshi Ishikawa, Masaru Takagi, Tomoaki Okimoto, Sigeyuki Murayama, Tetsuo Akimoto, Hitoshi Wada, Takeshi Arimura, Yoshitaka Sato, Masayuki Araya, Jun‐etsu Mizoe, Masahiko Gosho, Katsumasa Nakamura, Hiroki Shirato, Hideyuki Sakurai

**Affiliations:** ^1^ Department of Radiation Oncology Nagoya Proton Therapy Center Nagoya City West Medical Center Nagoya Japan; ^2^ Department of Radiology Nagoya City University Graduate School of Medical Sciences Nagoya Japan; ^3^ Department of Radiation Oncology Faculty of Medicine University of Tsukuba Tsukuba Japan; ^4^ Department of Radiation Oncology Sapporo Teishinkai Hospital Sapporo Japan; ^5^ Department of Radiology Hyogo Ion Beam Medical Center Tatsuno Japan; ^6^ Proton Therapy Division Shizuoka Cancer Center Hospital Nagaizumi Japan; ^7^ Division of Radiation Oncology and Particle Therapy National Cancer Center Hospital East Kashiwa Japan; ^8^ Department of Radiation Oncology Southern TOHOKU Proton Therapy Center Koriyama Japan; ^9^ Medipolis Proton Therapy and Research Center Ibusuki Japan; ^10^ Proton Therapy Center Fukui Prefectural Hospital Fukui Japan; ^11^ Proton Therapy Center Aizawa Hospital Matsumoto Japan; ^12^ Department of Clinical Trial and Clinical Epidemiology Faculty of Medicine University of Tsukuba Tsukuba Japan; ^13^ Department of Radiation Oncology Hamamatsu University School of Medicine Hamamatsu Japan; ^14^ Department of Radiation Medicine Hokkaido University Graduate School of Medicine Sapporo Japan

**Keywords:** Biochemical relapse‐free survival, late toxicity, multi‐institutional survey, prostate cancer, proton therapy

## Abstract

This is the first multi‐institutional retrospective survey of the long‐term outcomes of proton therapy (PT) for prostate cancer in Japan. This retrospective analysis comprised prostate cancer patients treated with PT at seven centers between January 2008 and December 2011 and was approved by each Institutional Review Board. The NCCN classification was used. Biochemical relapse was based on the Phoenix definition (nadir + 2.0 ng/mL). Toxicities were evaluated with the Common Terminology Criteria for Adverse Events version 4.0. There were 215, 520, and 556 patients in the low‐risk, intermediate‐risk, and high‐risk groups, respectively. The median follow‐up period of surviving patients was 69 months (range: 7–107). Among all patients, 98.8% were treated using a conventional fractionation schedule and 1.2% with a hypofractionation schedule; 58.5% and 21.5% received neoadjuvant and adjuvant androgen deprivation therapy, respectively. The 5‐year biochemical relapse‐free survival (bRFS) and overall survival rates in the low‐risk, intermediate‐risk, and high‐risk groups were 97.0%, 91.1%, and 83.1%, and 98.4%, 96.8%, and 95.2%, respectively. In the multivariate analysis, the NCCN classification was a significant prognostic factor for bRFS, but not overall survival. The incidence rates of grade 2 or more severe late gastrointestinal and genitourinary toxicities were 4.1% and 4.0%, retrospectively. This retrospective analysis of a multi‐institutional survey suggested that PT is effective and well‐tolerated for prostate cancer. Based on this result, a multi‐institutional prospective clinical trial (UMIN000025453) on PT for prostate cancer has just been initiated in order to define its role in Japan.

## Introduction

Prostate cancer is the second most common cancer in men with an annual estimated number of deaths of 300,000 worldwide 1. The number of patients with prostate cancer has also increased in Japan, and it was estimated to be the most common malignant tumor in men in 2016 2. A treatment algorithm based on staging and risk classification is used for localized prostate cancer, and surgery, radiotherapy, hormone therapy, and a multidisciplinary treatment combining these modalities are mainly performed. In a phase III study in which previous surgical treatment and radiotherapy for prostate cancer were compared, the outcomes of radiotherapy and total prostatectomy for early prostate cancer were found to be similar for the local control rate and bRFS 3, 4. Prostate cancer is one of the diseases treated with charged‐particle radiation therapy, such as proton and heavy‐ion beams, in many patients because treatment outcomes were improved by enhancing dose distributions based on experience with X‐ray radiation therapy and dose escalations on the assumption of this Ref. 5, 6, and high‐dose concentrations of charged‐particle beams were considered to be useful 7. In addition, the exposure dose of proton therapy (PT) is lower in the rectum and urinary bladder around the prostate than that of X‐ray irradiation applied at a similar dose 8, 9, for which reductions in adverse events may be expected. In a study by Loma Linda University, local PT at a total dose of 74 Gy equivalent (GyE) in 37 fractions was applied to 911 patients between 1991 and 1997; the 5‐year relapse‐free survival rate was 82%, and the incidence rates of grade 2 gastrointestinal (GI) and genitourinary (GU) toxicities were 3.5 and 5.4%, respectively, which were more favorable than the outcomes of X‐ray radiation therapy at that time 7, 10. In Japan, local PT was applied to 151 patients between 2004 and 2007 in a multicenter study involving three institutions, and the incidence rates of grade 2 GI and GU toxicities were 2.0 and 4.1%, respectively, showing a favorable outcome 11.

On the other hand, intensity‐modulated radiation therapy (IMRT) with external X‐ray irradiation has been widely performed. In physical studies, the irradiated volumes of the rectum and urinary bladder were smaller with PT 12, 13; however, differences in toxicities and quality of life (QOL) between PT and IMRT currently remain unclear. Sheets et al. 14 reported no significant differences in toxicities or QOL evaluations between PT and IMRT. Hoppe et al. 15 found significant differences in rectal urgency and frequent bowel movements, but not in other QOL scores between these two groups. Judgments and comparisons of the usefulness of IMRT are recommended for the application of PT to the treatment of prostate cancer, and the collection of evidence by prospective registration is considered desirable in the PT model policies issued by American Society for Radiation Oncology 16, indicating that the efficacy of PT remains controversial. In a systematic review, PT was not found to be cost‐effective for prostate cancer 17. In this study, the long‐term outcomes of patients who received PT at all seven institutions in Japan after 2008 were surveyed, with the aim of developing a new treatment strategy for the future.

## Materials and Methods

### Study design and patient eligibility

This was a retrospective analysis on prostate cancer treated with PT between January 2008 and December 2011 based on each institution's protocol decided by each Institutional Review Board (IRB) and was approved as a survey of the Japanese Radiation Oncology Study Group (JROSG2016‐R12). Seven centers in Japan were applicable during the surveyed period in this study, and this analysis was newly approved by each IRB. The host IRB number was 16‐04‐543‐24. Inclusion criteria were as follows: (1) histologically confirmed primary prostate cancer; (2) no lymph node and distant metastasis using computed tomography (CT) scans and bone scans; (3) Japanese men; (4) no prior pelvic radiotherapy; (5) hormone‐sensitive or hormone‐naive prostate cancer; (6) minimum follow‐up of 6 months for surviving patients; and (7) written informed consent. The NCCN classification was used for the risk categorization of prostate cancer. However, the very‐high‐risk group according to the NCCN criteria was included in the high‐risk group. Biochemical relapse was based on the Phoenix definition (nadir + 2.0 ng/mL). After PT, patients were followed up by regular studies including physical examinations and tumor marker evaluations. Prostate magnetic resonance imaging (MRI), pelvic CT scans, and bone scans were typically performed to evaluate distant metastases as well as the local tumor status, particularly when biochemical relapse was suspected or whenever necessary. The primary endpoint of this study was the 5‐year biochemical relapse‐free survival (bRFS). The secondary endpoints included the following: (1) 5‐year overall survival (OS); (2) 5‐year cause‐specific survival (CSS); (3) 5‐year bRF rates; (4) 5‐year clinical relapse‐free (cRF) rates; and (5) the incidence of grade 2 or more severe late GI and GU toxicities. bRFS was defined as the interval from the date of the final PT to the last follow‐up, biochemical relapse, or death date. OS was defined as the interval from the date of the final PT to the last follow‐up or death date. CSS was defined as the interval from the date of the final PT to the last follow‐up or death date relating to prostate cancer. BRF was defined as the interval from the date of the final PT to the last follow‐up, biochemical relapse, or clinical relapse. CRF was defined as the interval from the date of the final PT to the last follow‐up or clinical relapse. Predictive factors for bRFS, OS, and grade 2 or more severe late GI and GU toxicities were also evaluated using statistical analyses.

### Participating institutions

Seven institutions were equipped to provide PT during the periods of this study in Japan (National Cancer Center Hospital East, Kashiwa, Chiba; Shizuoka Cancer Center, Nagaizumi, Shizuoka; Hyogo Ion Beam Medical Center, Tatsuno, Hyogo; University of Tsukuba Faculty of Medicine, Tsukuba, Ibaraki; Southern TOHOKU Proton Therapy Center, Koriyama, Fukushima; Fukui Prefectural Hospital, Fukui, Fukui; and Medipolis Proton Therapy and Research Center, Ibusuki, Kagoshima).

### Treatment protocols and treatment systems

Proton therapy was delivered at a total dose of 70–80 GyE in 35–40 fractions (2 GyE/day, conventional fractionation) or 63–66 GyE in 21–22 fractions (3 GyE/day, hypofractionation). All irradiation doses were calculated at the center of the target volume. The accelerator complex consisted of a synchrotron (Mitsubishi Electric Corporation, Kobe, Japan, and Hitachi, Ltd., Tokyo, Japan) or a cyclotron (Sumitomo Heavy Industries, Ltd., Tokyo, Japan). Patients were treated with 210–235 MeV proton beams. Beam ranges were adjusted by a fine degrader. The spread‐out Bragg peaks (SOBP) of the proton were produced using bar‐ridge filters. Patient setup was performed daily by subtraction of the two sets of orthogonal digital radiographs or in‐room CT before each treatment. The translation and rotation of the patient detected by the positioning system were compensated for by adjustments to the treatment couch. The setup was continued until the bony landmarks and/or fiducial markers on digitally reconstructed radiographs agreed within 2 mm. Relative biological effectiveness (RBE) values for PT were set as 1.1. As all tissues are assumed to have almost the same RBE for PT, doses expressed in GyE were directly comparable to photon doses.

### Treatment planning

Radiation treatment plans were performed using a CT‐based three‐dimensional treatment planning system (FOCUS‐M, CMS, St. Louis, MO, Mitsubishi Electric Corporation, Kobe, Japan, and VQA, Hitachi, Ltd., Tokyo, Japan). Each patient was immobilized with a custom‐made thermoplastic cast, and 2‐ to 3‐mm‐thick CT images were taken under proper defecation and urination control. The clinical target volume (CTV) was defined as the prostate alone for low‐risk patients and as the prostate plus the proximal or whole seminal vesicles for intermediate‐risk and high‐risk patients. The planning target volume (PTV) consisted of the clinical target volume with optimal margins to account for uncertainties from the patient setup or internal organ motion, which were estimated at each institution (5–10 mm). Dose constraints for normal tissues were set on each institution's provision, which were based on the findings of a previous analysis 8. Bilateral opposed fields were used for proton dose delivery. A desirable treatment plan was defined as one that covered the PTV with 90% or more of the prescribed dose with or without the shrinking field technique. Therefore, treatment planning to encompass 95% of the CTV with 95% or more of the prescribed dose was sought. Doses were calculated based on the pencil beam algorithm. Adequate beam parameters, including beam energy, SOBP width, and degrader thickness, were assessed with FOCUS‐M or VQA, taking range uncertainty derived from PT into consideration.

### Statistical analysis

In comparison with the baseline clinical characteristics of the subgroups, Student's *t*‐test or Wilcoxon's rank‐sum test was used for continuous variables, and Fisher's exact test was used for categorical variables. bRFS, OS, CSS, and bRF rates were calculated using the Kaplan–Meier method. Differences between survival curves were examined by the log‐rank test. Hazard ratios and 95% confidence intervals (CIs) for bRFS, OS, and grade 2 or more severe late GI and GU toxicities were estimated using univariate and multivariate Cox's proportional hazards models. In the multivariate analysis, clinically meaningful covariates were selected from the candidates to avoid the multicollinearity of variables. A Fine–Gray competing risk analysis was also analyzed for OS. Missing data were excluded from the analysis, and the number is also listed in Table 1. Values of *P *< 0.05 were considered to be significant. All analyses were performed using SAS software version 9.4 (SAS Institute, Cary, NC). Toxicities were evaluated with the Common Terminology Criteria for Adverse Events version 4.0.

**Table 1:** Patient characteristics sorted by the NCCN risk classification

Variable	Level	Low	Intermediate	High	*P*	Total
Number	*N*	215	520	556		1291
Age	Mean ± SD	65 ± 7	67 ± 7	69 ± 7	<0.001	68 ± 7
	Median [Q1, Q3]^a^	65 [60, 71]	65 [60, 71]	65 [60, 71]	<0.001	68 [62, 73]
Performance status	0	208 (96.7)^b^	489 (94.0)	497 (89.4)	<0.001	1194 (92.5)
	1+	7 (3.3)	31 (6.0)	59 (10.6)		97 (7.5)
Operability	Operable	201 (93.5)	471 (90.6)	372 (66.9)	<0.001	1043 (80.8)
	Inoperable	14 (6.5)	49 (9.4)	185 (33.1)		248 (19.2)
T stage	1c‐2a	215 (100)	378 (72.7)	233 (42.1)	<0.001	826 (64.1)
	2b‐2c	0 (0)	142 (27.3)	125 (22.6)		267 (20.7)
	3a‐4	0 (0)	0 (0)	196 (35.3)		196 (15.2)
	Unknown	0	0	2 (‐)		2 (‐)
PSA value	Mean ± SD	6.1 ± 1.6	9.1 ± 4.1	24.5 ± 37.7	<0.001	15.3 ± 26.2
	Median [Q1,Q3]	6.0 [5.0, 7.1]	8.0 [5.7, 11.6]	13.4 [7.5, 26.5]	<0.001	8.5 [5.8, 14.8]
	<10	215 (100)	319 (61.6)	229 (41.2)	<0.001	763 (59.2)
	10–20	0 (0)	199 (38.4)	112 (20.1)		311 (24.1)
	20<	0 (0)	0 (0)	215 (38.7)		215 (16.7)
	Unknown	0	2 (‐)	0		2 (‐)
Gleason score	Mean ± SD	6.0 ± 0.0	6.8 ± 0.4	8.0 ± 0.9	<0.001	7.2 ± 1.0
	Median [Q1,Q3]	6 [6, 6]	7 [7, 7]	8 [7, 9]	<0.001	7 [6, 8]
	6	215 (100)	112 (21.5)	28 (5.1)	<0.001	355 (27.6)
	7	0 (0)	408 (78.5)	119 (21.5)		527 (40.9)
	8–10	0 (0)	0 (0)	406 (73.4)		406 (31.5)
	Unknown	0	0	3 (‐)		3 (‐)
ADT	No	180 (83.7)	276 (53.1)	67 (12.0)	<0.001	523 (40.5)
	Yes	35 (16.3)	244 (46.9)	489 (88.0)		768 (59.5)
ADT (pattern)	None	180 (83.7)	276 (53.1)	67 (12.0)	<0.001	523 (40.5)
	Neoadjuvant	28 (13.0)	188 (36.2)	275 (49.5)		491 (38.0)
	Adjuvant	2 (0.9)	2 (1)	9 (1.6)		13 (1.0)
	Neo + adjuvant	5 (2.4)	54 (10)	205 (36.9)		264 (20.5)
ADT period (only yes, month)	Mean ± SD	14 ± 17	13 ± 16	19 ± 19	<0.001	17 ± 18
	Median [Q1,Q3]	6 [4, 12]	6 [4, 14]	9 [6, 30]	<0.001	8 [6, 24]
ADT period (pattern 1, month)	0	180 (84.1)	276 (55.5)	67 (12.9)	<0.001	523 (42.6)
	0<, ≤6	19 (8.9)	119 (24.0)	176 (34.0)		314 (25.5)
	6<, <12	6 (2.8)	37 (7.4)	65 (12.5)		108 (8.8)
	12≤	9 (4.2)	65 (13.1)	210 (40.6)		284 (23.1)
ADT period 2 (pattern 2, month)	0	180 (84.1)	276 (55.5)	67 (12.9)	<0.001	523 (42.6)
	0<, ≤12	26 (12.2)	161 (32.4)	249 (48.1)		436 (35.5)
	12<, <24	2 (0.9)	27 (5.5)	59 (11.4)		88 (7.2)
	24≤	6 (2.8)	33 (6.6)	143 (27.6)		182 (14.8)
	Unknown	1 (‐)	23 (‐)	38 (‐)		62 (‐)
Diabetes mellitus	No	201 (93.5)	468 (90.0)	487 (87.6)	<0.001	1156 (89.5)
	Yes	14 (6.5)	52 (10.0)	69 (12.4)		135 (10.5)
Hypertension	No	173 (80.5)	378 (72.7)	422 (75.9)	<0.001	973 (75.4)
	Yes	42 (19.5)	142 (27.3)	134 (24.1)		318 (24.6)
Anticoagulant therapy	No	195 (90.1)	464 (91.0)	479 (86.2)	<0.001	1138 (88.1)
	Yes	20 (9.9)	56 (9.0)	77 (13.8)		153 (11.9)
Total dose (GyE)^c^	Mean ± SD	74 ± 1	74 ± 1	75 ± 2	<0.001	75 ± 1
	Median [Q1,Q3]	74 [74, 74]	74 [74, 74]	74 [74, 78]	<0.001	74 [74, 74]
Higher or lower dose^c^	≤74 GyE	208 (96.7)	493 (94.8)	405 (72.8)	<0.001	1106 (85.7)
	74 GyE <	7 (3.3)	27 (5.2)	151 (27.2)		185 (14.3)

NCCN, National Comprehensive Cancer Network; SD, standard deviation; PSA, prostate‐specific antigen; ADT, androgen deviation therapy.

^a^First and third quartiles. ^b^percent. ^c^The dose of hypofractionation was converted to conventional dose fractionation using a linear–quadratic model.

*P* values were calculated by Student's *t*‐test or Wilcoxon's rank‐sum test for continuous variables and Fisher's exact test for categorical variables.

## Results

### Patient and treatment characteristics

The total number of prostate cancer patients in all institutions during this period was 1,302, and 11 patients were excluded based on the above criteria: Six became unable to be followed up within 6 months, one received PT as re‐irradiation, two had bone metastasis from the beginning, and two were foreigners. Therefore, 1291 patients were analyzed in this study. Patient characteristics are summarized in Table 1. The PT protocol involving 70–80 GyE in 37–40 fractions (conventional fractionation, median; 74 GyE/37 Fr) and that involving 63–66 GyE in 20–21 fractions (hypofractionation) were delivered to 1274 (98.8%) and 17 (1.2%) patients, respectively. In total, 58.5% and 21.5% of patients received neoadjuvant (median, 6 months; range, 1–140 months) and adjuvant (median, 24 months; range, 1–90 months) ADT, respectively. More than 50% of intermediate‐risk patients and approximately 10% of high‐risk patients were not treated with ADT, while approximately 30% of intermediate‐risk patients and more than 50% of high‐risk patients received ADT for <1 year.

### Disease control and survival

**Figure 1:**
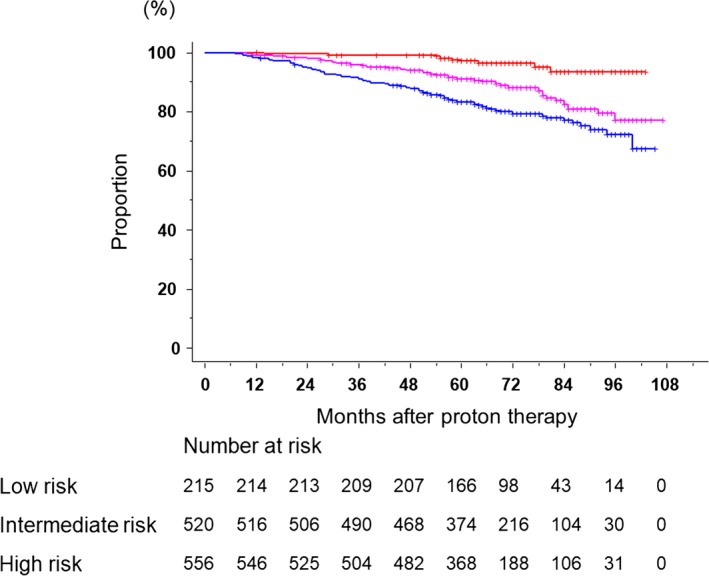
Biochemical relapse‐free survival according to the NCCN classification. Significant differences were observed in treatment results among the three groups (red line, low risk; pink line, intermediate risk; blue line, high risk).

**Figure 2:**
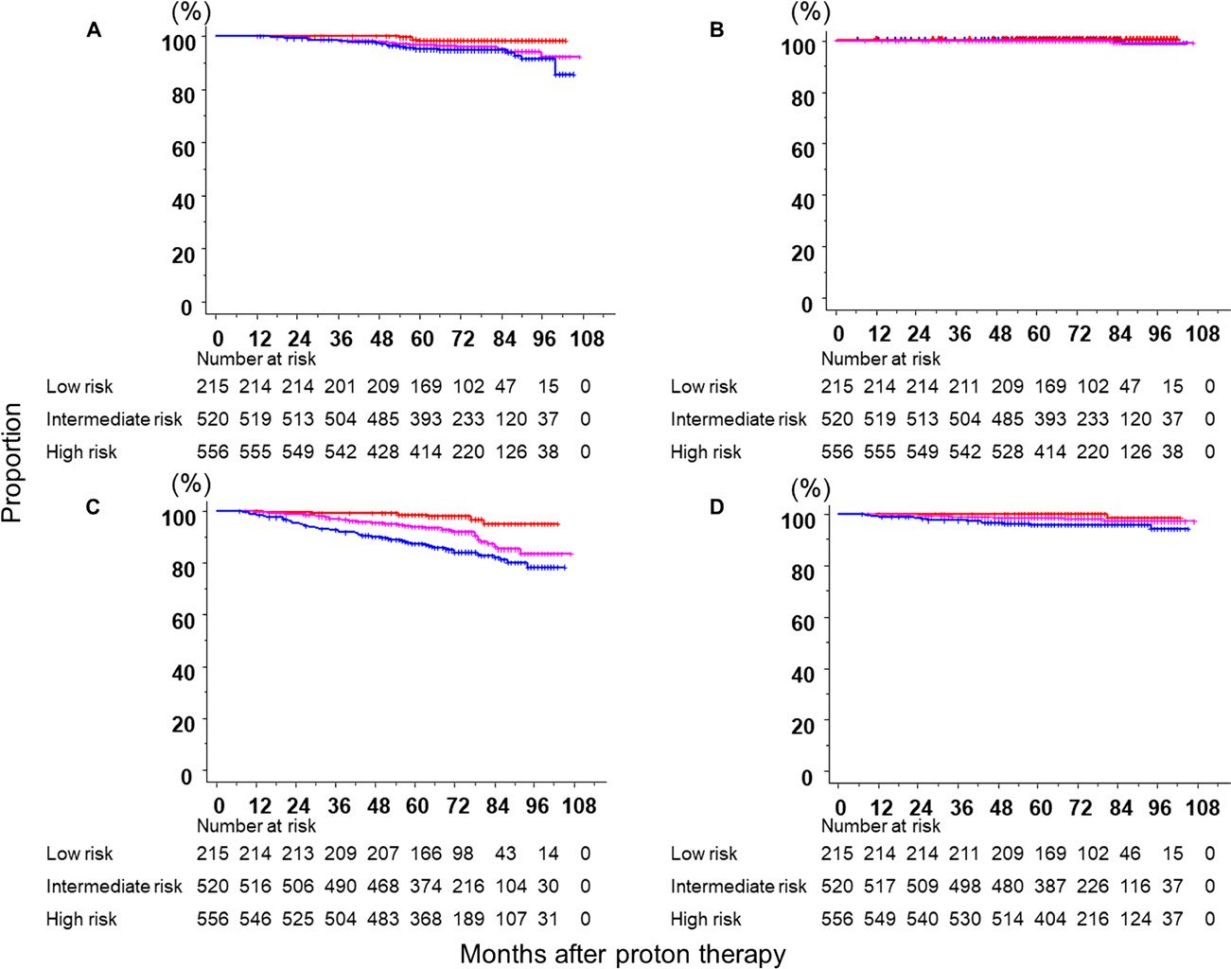
Overall survival (A), cause‐specific survival (B), biochemical relapse‐free (C), and clinical relapse‐free curves (D) according to the NCCC group (red line, low risk; pink line, intermediate risk; blue line, high risk).

The median follow‐up period of surviving patients was 69 months (range: 7–107). Figure 1 shows bRFS according to the NCCN classification. Five‐year bRFS rates in the low‐risk, intermediate‐risk, and high‐risk groups were 97.0% (95% CI; 93.4–98.6%), 91.0% (88.2–93.2%), and 83.1% (79.8–86.1%), respectively. Significant differences were observed in treatment results among the three groups. Figure 2 (A‐D) shows OS, CSS, bRF, and cRF according to the NCCC group, and a summary of disease control and survival rates is provided in Table 2. Biochemical relapse was observed in 137 patients, 35 of whom showed clinical relapse. Local recurrence was noted in nine patients. Twelve patients developed lymph node metastases. In addition, 17 patients developed distant bone or lung metastases. Fifty‐seven patients died, and 53 of them died of other diseases.

**Table 2:** Summary of disease control and survival data

Outcome	Group	Number of events (%)	Survival and control rates at 5 years (95% CI)
bRFS	Low risk	9 (4.2)	97.0 (93.4, 98.6)
	Intermediate risk	67 (12.9)	91.0 (88.2, 93.2)
	High risk	113 (20.3)	83.2 (79.8, 86.1)
OS	Low risk	3 (1.4)	98.4 (95.2, 99.5)
	Intermediate risk	22 (4.2)	96.8 (94.9,98.0)
	High risk	32 (5.8)	95.2 (93.0,96.7)
CSS	Low risk	0 (0.0)	100 (‐)
	Intermediate risk	1 (0.2)	100 (‐)
	High risk	3 (0.5)	99.6 (98.5, 99.9)
bRF	Low risk	6 (2.8)	98.6 (95.6, 99.5)
	Intermediate risk	47 (9.0)	93.9 (91.4, 95.7)
	High risk	84 (15.1)	87.4 (84.3, 89.9)
cRF	Low risk	1 (0.5)	100 (‐)
	Intermediate risk	11 (2.1)	98.2 (96.6, 99.1)
	High risk	23 (4.1)	95.9 (93.9, 97.3)

CI, confidence interval; bRFS, biochemical relapse‐free survival; OS, overall survival; CSS, cause‐specific survival; bRF, biochemical relapse‐free; cRF, clinical relapse‐free.

Table 3 summarizes the results of univariate analyses on various factors associated with bRFS and OS. The NCCN classification, age, performance status, operability, T stage, Gleason score, PSA value, and ADT were associated with bRFS in the univariate analysis. A multivariate analysis was performed using clinical factors selected by the univariate analysis (Table 3) with a *P* value of <0.05 for each outcome (i.e., bRFS and OS). More important factors in this study were selected to avoid the multicollinearity of variables in the multivariate analysis. The T stage, PSA, and Gleason score were excluded from the multivariate analysis because NCCN risk groups were categorized based on these factors. In addition, the ADT period was excluded from the multivariate model because it strongly correlated with the use of ADT. According to the multivariate analysis, the NCCN classification was a significant prognostic factor for bRFS, but not for OS (Table 4). In addition, we were unable to apply the Fine–Gray competing risk model to this dataset because there were only four deaths from prostate cancer.

**Table 3:** Univariate Cox analysis for disease control and survival

Outcome	Factor	*N* of data	*N* of events (%)	Level	Unadjusted HR (95% CI)	*P*	*P* for the global test
bRFS	NCCN risk group	1291	189 (14.6)	Intermediate vs. low	3.23 (1.61, 6.47)	<0.001	<0.001
				High vs. low	5.39 (2.73, 10.62)	<0.001	
				High vs. intermediate	1.67 (1.23, 2.26)	<0.001	
	Age	1291	189 (14.6)	Increase of 10 years	1.28 (1.04, 1.57)	0.021	0.021
	Performance status	1291	189 (14.6)	1≤ vs. 0	2.37 (1.59, 3.55)	<0.001	<0.001
	Operability	1291	189 (14.6)	Inoperable vs. operable	1.96 (1.44, 2.67)	<0.001	<0.001
	T stage	1289	188 (14.6)	2b‐2c vs. 1c‐2a	1.50 (1.06, 2.14)	0.024	<0.001
				3a≤ vs. 1c‐2a	2.18 (1.54, 3.10)	<0.001	
	PSA	1289	188 (14.6)	10–20 vs. <10	1.98 (1.42, 2.78)	<0.001	<0.001
				20< vs. <10	2.34 (1.64, 3.34)	<0.001	
	Gleason score	1288	188 (14.6)	7 vs. 6	2.34 (1.47, 3.73)	<0.001	<0.001
				8–10 vs. 6	3.78 (2.39, 5.98)	<0.001	
	ADT	1291	189 (14.6)	Yes vs. No	1.58 (1.16, 2.15)	0.004	0.004
	ADT period	706	122 (17.3)	Increase of 10 months	1.00 (0.91, 1.10)	0.94	0.94
	ADT period (pattern 1, month)	1229	180 (14.6)	0<, ≤6 vs. 0	1.90 (1.33, 2.70)	<0.001	0.005
				6<, <12 vs. 0	1.29 (0.74, 2.24)	0.37	
				12≤ vs. 0	1.33 (0.89, 1.99)	0.16	
	ADT period (pattern 2, month)	1229	180 (14.6)	0<, ≤12 vs. 0	1.70 (1.21, 2.38)	0.002	0.022
				12<, <24 vs. 0	1.27 (0.68, 2.36)	0.45	
				24≤ vs. 0	1.42 (0.90, 2.22)	0.13	
	Total dose (GyE)^a^	1291	189 (14.6)	Increase of 10 GyE	0.52 (0.17, 1.59)	0.25	0.25
	Higher or lower dose	1291	189 (14.6)	74 GyE < vs. ≤ 74 GyE	0.77 (0.49, 1.23)	0.28	0.28
OS	Group	1291	57 (4.4)	Intermediate vs. low	3.12 (0.93, 10.42)	0.065	0.045
				High vs. low	4.25 (1.30, 13.89)	0.017	
				High vs. intermediate	1.36 (0.79, 2.35)	0.26	
	Age	1291	57 (4.4)	Increase of 10 years	2.64 (1.77, 3.95)	<0.001	<0.001
	Performance status	1291	57 (4.4)	1≤ vs. 0	3.62 (1.91, 6.85)	<0.001	<0.001
	Operability	1291	57 (4.4)	Inoperable vs. operable	2.46 (1.43, 4.22)	0.001	0.001
	T stage	1289	57 (4.4)	2b‐2c vs. 1c‐2a	1.08 (0.56, 2.10)	0.81	0.48
				3a≤ vs. 1c‐2a	1.51 (0.78, 2.92)	0.22	
	PSA	1289	56 (4.3)	10–20 vs. <10	1.35 (0.72, 2.52)	0.35	0.28
				20< vs. <10	1.67 (0.86, 3.22)	0.13	
	Gleason score	1288	56 (4.3)	7 vs. 6	2.79 (1.22, 6.40)	0.015	0.043
				8–10 vs. 6	2.71 (1.15, 6.37)	0.023	
	ADT	1291	57 (4.4)	Yes vs. No	1.90 (1.06, 3.43)	0.032	0.032
	ADT period	706	40 (5.7)	Increase of 10 months	0.95 (0.80, 1.14)	0.61	0.61
	ADT period (pattern 1, month)	1229	55 (4.5)	0<, ≤6 vs. 0	2.41 (1.25, 4.64)	0.009	0.075
				6<, <12 vs. 0	1.55 (0.56, 4.27)	0.40	
				12≤ vs. 0	1.60 (0.76, 3.37)	0.21	
	ADT period (pattern 2, month)	1229	55 (4.5)	0<, ≤12 vs. 0	2.20 (1.17, 4.11)	0.014	0.094
				12<, <24 vs. 0	1.18 (0.34, 4.08)	0.79	
				24≤ vs. 0	1.73 (0.76, 3.96)	0.19	
	Total dose (GyE)^a^	1291	57 (4.4)	Increase of 10 GyE	0.39 (0.05, 3.22)	0.39	0.39
	Higher or lower dose	1291	57 (4.4)	74 GyE < vs. ≤ 74 GyE	0.63 (0.25, 1.58)	0.33	0.33

*N*, number; HR, hazard ratio; CI, confidence interval; bRFS, biochemical relapse‐free survival; NCCN, National Comprehensive Cancer Network; vs., versus; PSA, prostate‐specific antigen; ADT, androgen deviation therapy; OS, overall survival.

a The dose of hypofractionation was converted to conventional dose fractionation using a linear–quadratic model.

The global test is an assessment of whether a factor is significant.

**Table 4:** Multivariate Cox analysis for disease control and survival

Outcome	Factor	Adjusted HR (95% CI)	*P*
bRFS	NCCN risk group, intermediate vs. low	3.24 (1.60, 6.56)	0.001
	NCCN risk group, high vs. low	5.04 (2.42, 10.48)	<0.001
	NCCN risk group, high vs. intermediate	1.55 (1.09, 2.20)	0.014
	Age, Increase of 10 years	1.04 (0.84, 1.30)	0.70
	Performance status, 1≤ vs. 0	1.87 (1.23, 2.85)	0.003
	Operability, inoperable vs. operable	1.42 (1.01, 1.99)	0.045
	ADT, Yes vs. No	0.85 (0.59, 1.23)	0.39
OS	NCCN risk group, intermediate vs. low	2.57 (0.75, 8.74)	0.13
	NCCN risk group, high vs. low	2.58 (0.72, 9.30)	0.15
	NCCN risk group, high vs. intermediate	1.01 (0.54, 1.87)	0.99
	Age, increase of 10 years	2.07 (1.36, 3.16)	<0.001
	Performance status, 1≤ vs. 0	2.03 (1.02, 4.03)	0.043
	Operability, inoperable vs. operable	1.50 (0.83, 2.72)	0.18
	ADT, Yes vs. No	1.07 (0.54, 2.12)	0.85

HR, hazard ratio; CI, confidence interval; bRFS, biochemical relapse‐free survival; NCCN, National Comprehensive Cancer Network; vs., versus; ADT, androgen deviation therapy; OS, overall survival.

### Complications

**Figure 3:**
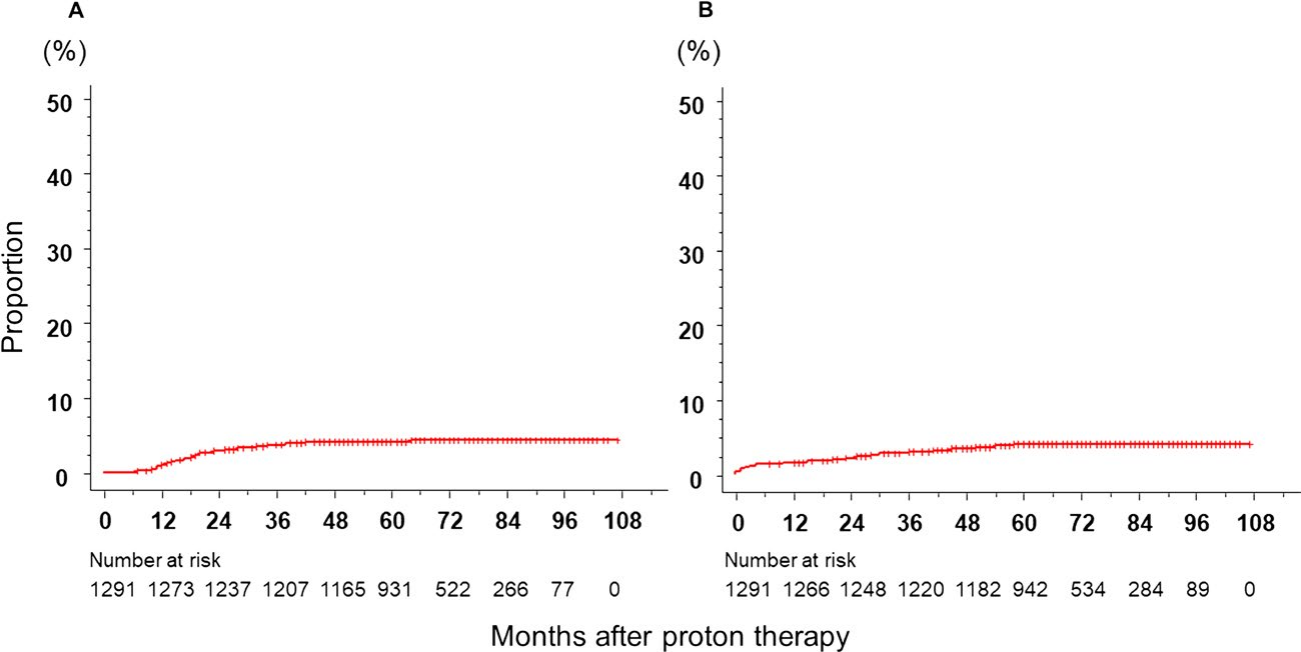
Cumulative incidence rates of the late grade 2 or more severe gastrointestinal toxicity curve (A) and late genitourinary toxicity curve (B).

The incidence rates of grade 2 or more severe late GI and GU toxicities were 4.1% (3.1–5.3%) and 4.0% (3.1–5.3%), respectively. Grade 3 GI and GU toxicities were only observed in six (0.5%) and four (0.3%) patients, respectively. **Figure** 3 shows the cumulative incidence rates of grade 2 or more severe GI and GU toxicities curves. Table 5 summarizes the results of univariate analyses on various factors associated with grade 2 or more severe late GI and GU toxicities. According to the univariate analysis for GU, significant differences were observed for age, operability, PSA, ADT, and dose escalations, whereas no significant differences were noted in the multivariate analysis.

**Table 5:** Univariate Cox analysis for toxicities

Outcome	Factor	*N* of data	*N* of events (%)	Level	Unadjusted HR (95% CI)	*P*	*P* for the global test
GU^a^	NCCN risk group	1291	52 (4.0)	Intermediate vs. low	1.39 (0.60, 3.24)	0.44	0.74
				High vs. low	1.24 (0.53, 2.90)	0.62	
				High vs. intermediate	0.89 (0.50, 1.60)	0.69	
	Age	1291	52 (4.0)	Increase of 10 years	1.68 (1.12, 2.50)	0.011	0.011
	Performance status	1291	52 (4.0)	1≤ vs. 0	0.78 (0.24, 2.49)	0.67	0.67
	Operability	1291	52 (4.0)	Inoperable vs. operable	2.13 (1.20, 3.81)	0.010	0.010
	T stage	1289	52 (4.0)	2b‐2c vs. 1c‐2a	1.10 (0.55, 2.18)	0.79	0.68
				3a≤ vs. 1c‐2a	1.37 (0.67, 2.80)	0.38	
	PSA	1289	52 (4.0)	10–20 vs. <10	2.06 (1.10,3.83)	0.023	0.043
				20< vs. <10	1.97 (0.97, 3.97)	0.060	
	Gleason score	1288	52 (4.0)	7 vs. 6	1.23 (0.64, 2.37)	0.53	0.49
				8–10 vs. 6	0.82 (0.39, 1.75)	0.61	
	ADT	1291	52 (4.0)	Yes vs. No	2.31 (1.21, 4.41)	0.011	0.011
	ADT period	706	34 (4.8)	Increase of 10 months	1.21 (1.07, 1.37)	0.002	0.002
	ADT period (pattern 1, month)	1229	180 (14.6)	0<, ≤6 vs. 0	0.84 (0.31, 2.23)	0.72	<0.001
				6<, <12 vs. 0	2.04 (0.72, 5.78)	0.18	
				12≤ vs. 0	3.64 (1.81, 7.31)	<0.001	
	ADT period (pattern 2, month)	1229	46 (3.7)	0<, ≤12 vs. 0	1.31 (0.60, 2.87)	0.50	0.002
				12<, <24 vs. 0	2.97 (1.11, 7.90)	0.030	
				24≤ vs. 0	3.75 (1.76, 8.01)	<0.001	
	Diabetes mellitus	1291	52 (4.0)	Yes vs. No	0.17 (0.02, 1.20)	0.076	0.076
	Hypertension	1291	52 (4.0)	Yes vs. No	0.92 (0.02, 1.20)	0.80	0.80
	Anticoagulant therapy	1291	52 (4.0)	Yes vs. No	1.16 (0.52, 2.57)	0.72	0.72
	Total dose (GyE)^b^	1291	52 (4.0)	Increase of 10 GyE	3.26 (0.64, 16.53)	0.15	0.15
	Higher or lower dose^b^	1291	52 (4.0)	74 GyE < vs. ≤ 74 GyE	2.03 (1.08, 3.81)	0.027	0.027
GI^a^	Group	1291	53 (4.1)	Intermediate vs. low	0.97 (0.44, 2.11)	0.93	1.00
				High vs. low	0.99 (0.46, 2.14)	0.98	
				High vs. intermediate	1.02 (0.57, 1.85)	0.94	
	Age	1291	53 (4.1)	Increase of 10 years	1.45 (0.98, 2.14)	0.063	0.063
	Performance status	1291	53 (4.1)	1≤ vs. 0	1.35 (0.54, 3.38)	0.53	0.53
	Operability	1291	53 (4.1)	Inoperable vs. operable	0.88 (0.43, 1.80)	0.72	0.72
	T stage	1289	53 (4.1)	2b‐2c vs. 1c‐2a	0.55 (0.25, 1.23)	0.15	0.31
				3a≤ vs. 1c‐2a	0.75 (0.34, 1.67)	0.48	
	PSA	1289	53 (4.1)	10–20 vs. <10	1.07 (0.56, 2.06)	0.83	0.89
				20< vs. <10	1.19 (0.58, 2.44)	0.63	
	Gleason score	1288	53 (4.1)	7 vs. 6	0.90 (0.46, 1.76)	0.76	0.88
				8–10 vs. 6	1.06 (0.53, 2.10)	0.87	
	ADT	1291	53 (4.1)	Yes vs. No	0.82 (0.48, 1.40)	0.46	0.46
	ADT period	706	26 (3.7)	Increase of 10 months	0.90 (0.70, 1.15)	0.40	0.40
	ADT period (pattern 1, month)	1229	50 (4.1)	0<, ≤6 vs. 0	0.83 (0.42, 1.66)	0.60	0.56
				6<, <12 vs. 0	1.19 (0.49, 2.91)	0.71	
				12≤ vs. 0	0.61 (0.27, 1.35)	0.22	
	ADT period (pattern 2, month)	1229	50 (4.1)	0<, ≤12 vs. 0	0.89 (0.48, 1.64)	0.71	0.57
				12<, <24 vs. 0	0.99 (0.34, 2.86)	0.99	
				24≤ vs. 0	0.47 (0.16, 1.35)	0.16	
	Diabetes mellitus	1291	53 (4.1)	Yes vs. No	0.70 (0.25, 1.94)	0.50	0.50
	Hypertension	1291	53 (4.1)	Yes vs. No	1.34 (0.75, 2.41)	0.33	0.33
	Anticoagulant therapy	1291	53 (4.1)	Yes vs. No	1.36 (0.64, 2.89)	0.42	0.42
	Total dose (GyE)^b^	1291	53 (4.1)	Increase of 10 GyE	0.36 (0.04, 2.88)	0.33	0.33
	Higher or lower dose^b^	1291	53 (4.1)	74 GyE < vs. ≤ 74 GyE	0.48 (0.17, 1.33)	0.16	0.16

*N*, number; HR, hazard ratio; CI, confidence interval; GU, genitourinary; NCCN, National Comprehensive Cancer Network; vs., versus; PSA, prostate‐specific antigen; ADT, androgen deviation therapy; GI, gastrointestinal.

^a^Grade 2 or more severe. ^b^The dose of hypofractionation was converted to conventional dose fractionation using a linear–quadratic model.

The global test is an assessment of whether a factor is significant.

## Discussion

The present study is the first retrospective analysis on a multi‐institutional survey in Japan to evaluate the effectiveness and feasibility of PT on prostate cancer. In an analysis of 1291 patients during a median follow‐up period greater than 5 years, 5‐year bRFS rates were 97.0, 91.1, and 83.1% in the low‐risk, intermediate‐risk, and high‐risk groups, respectively, and 5‐year OS rates were 98.4, 96.8, and 95.2%, respectively. The incidence of grade 2 or more severe adverse events was lower than 5% in all groups. These results were consistent with previous findings reported by Bryant et al. 18. Although the present study had the limitation of being a retrospective survey, such that it was impossible to ascertain details of the dose–volume histogram in each case, only a few large‐scale studies on PT for prostate cancer obtaining long‐term outcomes have been performed, and this was the initial survey in Japan. Therefore, the present results may provide important information for the future development of PT.

We investigated whether the NCCN risk classification is an independent prognostic factor for bRFS and OS. As shown in Tables 3 and 4, it was a prognostic factor for bRFS, whereas verification for OS was not possible. Univariate and multivariate analyses revealed several factors associated with bRFS and OS other than the NCCN risk groups, that is, age, operability, and performance status. However, only PS was commonly significant for bRFS and OS, and this may have been due to the influence of a small number of deaths from prostate cancer (four patients) despite 40% of all patients being included in the high‐risk group. The total dose showed no significant difference for all endpoints when analyzed with a continuous variable or in a dichotomous group. However, the risk seemed to decrease as the dose increased.

Although a combination with ADT is a well‐known prognostic factor associated with biochemical relapse 19, 20, 21, the irradiation dose was approximately 70 Gy in these studies; the contribution of high‐dose irradiation to bRFS and OS currently remains unknown. In the present study, as irradiation was applied at 70–80 Gy (median: 74 GyE delivered in 37 fractions) including the high‐risk group, the contribution of ADT to bRFS and OS was unclear in the multivariate analysis. Even when only the intermediate‐risk or high‐risk group was included in the analysis, the CI of the hazard ratio was wide due to the small number of events and ADT performed in most patients, which is inappropriate for statistical analyses. Thus, a retrospective analysis of the additional effects of ADT for PT and optimum combination periods was difficult in the present study.

Patients at very high risk were analyzed as those at high risk, and this was another limitation of the present study. This was due to insufficient information on the positive core number. However, the overall 5‐year bRFS rate was 83.1% in the high‐risk group including very‐high‐risk patients, whereas that of patients treated with neoadjuvant + adjuvant long‐term ADT was 37%, suggesting that the outcome was favorable. As the median follow‐up period was only 69 months, longer course observations may be necessary for the final evaluation of OS from prostate cancer.

In the past two decades, IMRT for localized prostate cancer has been spreading worldwide. Favorable outcomes with IMRT for prostate cancer have accumulated, and IMRT with or without ADT is becoming an indispensable treatment modality for patients who refuse surgery or are medically inoperable. An alternative to or theoretically better treatment option than IMRT is PT, and a comparison among these treatment modalities needs to be performed for physical dose distributions and biological effectiveness 22, 23. Previous planning studies comparing PT and IMRT suggested the benefits of reducing the dosage of the surrounding OARs of PT over IMRT within the low‐ to medium‐dose range of radiation rather than the high‐dose range 12. In the present study, the incidence rates of grade 2 or more severe adverse events of the GI and GU were lower than 5%. Representative results for IMRT and PT for prostate carcinoma are shown in Table 6 10, 18, 24, 25, 26, 27, 28. The passive method and bone reconstruction were employed for the irradiation method in PT in all and more than 90% of cases, respectively. The internal prostate motion was 4–7 mm, being non‐negligible, as reported by Bylund 29 and Frank et al. 30. The setup error of internal motion may be canceled by marker verification, which reduces the lateral margins of the rectum and urinary bladder. Furthermore, rectal and urinary bladder irradiation volumes may be reduced using the scanning technique 31, 32. Therefore, more favorable outcomes than those achieved in the present study are expected in the future.

**Table 6:** Representative reported results of radical radiotherapy for prostate cancer

Author, Year	Patient number	Modality	Dose fractionation (Gy or GyE)	5‐year bRFS for low, intermediate, and high and very high risk	GI late toxicity; ≥ grade 2 and ≥ grade 3 (%)	GU late toxicity; ≥ grade 2 and ≥ grade 3 (%)	Median follow‐up (months)
Zelefsky, et al. 2006 24	561	IMRT	81 Gy/45 Fr	85%, 76%, and 72% (8 year)	1.8% and 0.1%	12% and 3%	84
Guckenberger, et al. 2014 25	150	IMRT	73.9–76.2 Gy/32–33 Fr	88%, 80%, and 78% (D'Amico classification)	4.8% and 1.3%	22.4% and 3.8%	50
Kupelian, et al. 2005 26	100	IMRT	70 Gy/28 Fr	97%, 93%, and 75% (D'Amico classification)	10% and 3%	12% and 1%	66
Dearnaley, et al. 2016 27	1065	IMRT	74 Gy/37 Fr	82.3% (all)	13.7% and 2%	9.1% and 3%	62.6
	1074	IMRT	60 Gy/20 Fr	85.3% (all)	12.0% and 3%	11.7% and 6%	62.2
	1077	IMRT	57 Gy/19 Fr	80.1% (all)	11.2% and 4%	6.6% and 3%	62.4
Bryant, et al. 2016 18	1327	Proton	74–82 GyE/37–41 Fr	bRFS; NR bRF; 98.9%, 93.9%, and 74.0%	NR and 0.6%	NR and 2.9%	66
Schulte, et al. 2000 10	911	Proton	74–75 GyE/37–40 Fr	82% (all)	3.5% and 0%	5.4% and 0%	39
Mendenhall, et al. 2014 28	211	Proton	78–82 GyE/39–41 Fr	bRFS; NR bRF; 99%, 99%, and 76%	NR and 1.0%	NR and 0.9%	62
This study, 2017	1291	Proton	70–80 GyE/ 35–40 Fr, 63–66 GyE/ 21–22 Fr	97.0%, 91.0%, and 83.1%	4.1% and 0.5%	4.0% and 0.3%	69

bRFS, biochemical relapse‐free survival; GI, gastrointestinal; GU, genitourinary; IMRT, intensity‐modulated radiation therapy; bRF, biochemical relapse‐free; NR, not reported.

Quality of life evaluations and cost‐effectiveness are important when comparing radiation therapies for prostate cancer, but were not surveyed herein. In the study by Sheets et al. 14, no significant differences were observed in toxicity or QOL. However, as a limit of the analysis from the database, there were miscellaneous doses, mixed examples of X‐rays combined, and insufficient matching. Therefore, the accumulation of more data is necessary. Hoppe et al. 15 surveyed QOL evaluations of patients who received IMRT and PT using EPIC. No significant differences were noted in the total score between the two groups; however, rectal urgency (*P *= 0.02) and frequent bowel movements (*P *= 0.05) were favorable in the PT group, and this may have being due to the rectal dose being reduced in PT, whereas it was not possible to reduce the urethral dose. Based on these findings, PARTIQoL (NCT01617161) is being conducted in the United States and an interim analysis is scheduled for 2018 33.

The biggest issue associated with PT is cost. Verma et al. conducted a systematic review and showed that cost‐effectiveness for prostate cancer treated with PT is suboptimal 17. If the cost of PT is sufficiently reduced, it may become more widely performed even though differences in its effects and adverse events from those of IMRT are small. One solution to achieve favorable cost‐benefit performance while sufficiently utilizing the physical advantage of PT may be the introduction of hypofractionation. Although the use of a linear–quadratic model is controversial for conversion to hypofractionation 34, 35, similar to radiobiology, hypofractionation is theoretically advantageous for prostate cancer and also needs to be considered for PT in order to reduce the burden on patients. The mean *α*/*β* value of prostate cancer was previously reported to be approximately 1.5–3.1 Gy, which is very low 36, 37, and the potential doubling time was 30 days or longer, which is very long 38. When dose fractionations of 70 Gy/28 Fr and 63 Gy/21 Fr were compared with 78 Gy/39 Fr on the assumption that the *α*/*β* value of prostate cancer is 1.5 Gy and that of late rectal disorders is 3.0 Gy, 2 Gy‐converted effects were 80 and 81 Gy, being similar or higher, while those of the rectal dose were 77 and 75.6 Gy, being theoretically reduced. However, the incidence of adverse events induced by X‐ray IMRT hypofractionation was unexpectedly high in several studies 26, 27. Therefore, hypofractionated PT may exert efficacy by reducing the irradiation volume of the organs at risk to less than that in IMRT. A clinical study on hypofractionated PT has been initiated, such as that reported by Henderson et al. 39.

In conclusion, this retrospective analysis of a multi‐institutional survey suggested that PT is effective and tolerated well for prostate cancer. However, further evidence for the effectiveness of PT for prostate cancer is needed. Based on the present results, a multi‐institutional prospective clinical trial involving all participating Japanese PT centers has just been started in order to evaluate the efficacy, toxicities, QOL, and cost‐effectiveness of PT and define its role in the treatment of prostate cancer in Japan (UMIN000025453). The data obtained will be compared to those from a large cohort registered to the Japanese Radiation Oncology Study Group in the future.

## Conflict of Interest

None declared.
